# Peroxisomal Proteome Mining of Sweet Pepper (*Capsicum annuum* L.) Fruit Ripening Through Whole Isobaric Tags for Relative and Absolute Quantitation Analysis

**DOI:** 10.3389/fpls.2022.893376

**Published:** 2022-05-09

**Authors:** Salvador González-Gordo, José M. Palma, Francisco J. Corpas

**Affiliations:** Group of Antioxidants, Free Radicals and Nitric Oxide in Biotechnology, Food and Agriculture, Department of Biochemistry, Cell and Molecular Biology of Plants, Estación Experimental del Zaidín, Spanish National Research Council (CSIC), Granada, Spain

**Keywords:** iTRAQ, phenylpropanoids, pepper fruit, ripening, jasmonic acid, peroxisomes, proteome, sulfite oxidase

## Abstract

Peroxisomes are ubiquitous organelles from eukaryotic cells characterized by an active nitro-oxidative metabolism. They have a relevant metabolic plasticity depending on the organism, tissue, developmental stage, or physiological/stress/environmental conditions. Our knowledge of peroxisomal metabolism from fruits is very limited but its proteome is even less known. Using sweet pepper (*Capsicum annuum* L.) fruits at two ripening stages (immature green and ripe red), it was analyzed the proteomic peroxisomal composition by quantitative isobaric tags for relative and absolute quantitation (iTRAQ)-based protein profiling. For this aim, it was accomplished a comparative analysis of the pepper fruit whole proteome obtained by iTRAQ versus the identified peroxisomal protein profile from *Arabidopsis thaliana*. This allowed identifying 57 peroxisomal proteins. Among these proteins, 49 were located in the peroxisomal matrix, 36 proteins had a peroxisomal targeting signal type 1 (PTS1), 8 had a PTS type 2, 5 lacked this type of peptide signal, and 8 proteins were associated with the membrane of this organelle. Furthermore, 34 proteins showed significant differences during the ripening of the fruits, 19 being overexpressed and 15 repressed. Based on previous biochemical studies using purified peroxisomes from pepper fruits, it could be said that some of the identified peroxisomal proteins were corroborated as part of the pepper fruit antioxidant metabolism (catalase, superoxide dismutase, ascorbate peroxidase, monodehydroascorbate reductase, dehydroascorbate reductaseglutathione reductase, 6-phosphogluconate dehydrogenase and NADP-isocitrate dehydrogenase), the β-oxidation pathway (acyl-coenzyme A oxidase, 3-hydroxyacyl-CoA dehydrogenase, enoyl-CoA hydratase), while other identified proteins could be considered “new” or “unexpected” in fruit peroxisomes like urate oxidase (UO), sulfite oxidase (SO), 5-methyltetrahydropteroyltriglutamate-homocysteine methyltransferase (METE1), 12-oxophytodienoate reductase 3 (OPR3) or 4-coumarate-CoA ligase (4CL), which participate in different metabolic pathways such as purine, sulfur, L-methionine, jasmonic acid (JA) or phenylpropanoid metabolisms. In summary, the present data provide new insights into the complex metabolic machinery of peroxisomes in fruit and open new windows of research into the peroxisomal functions during fruit ripening.

## Introduction

Plant peroxisomal metabolism shares common enzymatic components with peroxisomes from other eukaryotic organisms. However, the metabolic plasticity of plant peroxisomes differs depending on the species, organ (root, leaf, cotyledon, flower, and fruit), stage of development, or environmental conditions (Hu et al., 2012; Reumann and Bartel, 2016; Pan et al., 2020; Huang et al., 2022). This indicates that the protein and metabolic peroxisomal profiles are diverse being a good example of the metabolic changes occurring from peroxisomes present in cotyledons (called glyoxysomes) to leaf-type peroxisomes (Goto-Yamada et al., 2015). As reflected in their name, peroxisomes are defined as subcellular compartments with a high content of hydrogen peroxide (H_2_O_2_) which is generated by different oxidase enzymes and contain catalase as the key and major enzyme to control the potential oxidative damage triggered by the overproduction of these reactive oxygen species (ROS) (Corpas et al., 2019, 2020).

Sweet pepper (*Capsicum annuum* L.) fruits are one of the horticultural crops of the greatest agronomic importance due to their worldwide consumption. The fruits are characterized by having a high content of vitamin C and A (Palma et al., 2015, 2020b). Pepper fruits have an ethylene-independent ripening and therefore it is considered a non-climacteric fruit. During ripening, pepper fruits undergo drastic biochemical and phenotypical changes, being the color shift from green to red the most evident since chloroplasts are transformed into chromoplasts as a consequence of the chlorophyll degradation and the biosynthesis of carotenoids, xanthophylls, and anthocyanins. In previous studies, it has been analyzed the sweet pepper fruit ripening at biochemical and molecular levels including the transcriptome (González-Gordo et al., 2019), as well as the metabolism of NADPH-generating enzymes (Mateos et al., 2009; Muñoz-Vargas et al., 2018, 2020), ROS metabolism (Rodríguez-Ruiz et al., 2017a,^2019^; Chu-Puga et al., 2019; González-Gordo et al., 2020, 2022a; Palma et al., 2020a), reactive nitrogen species (Chaki et al., 2015; Rodríguez-Ruiz et al., 2017b), the metabolome (Guevara et al., 2021), and the plastidial and mitochondrial proteomes (Rödiger et al., 2021; González-Gordo et al., 2022b). However, little is known about the proteome from peroxisomes and how it can be modulated during ripening. In a preliminary study, using pepper fruit peroxisomes purified by the combination of differential and sucrose density-gradient centrifugations and then subjected to 2-D electrophoresis and MALDI-TOF/TOF analyses, it was possible to discriminate 39 polypeptides, but only 13 of them were identified with a high protein score (99%) confidence interval (C.I.) (Palma et al., 2018). In the present study, an alternative and powerful approach has been used, the quantitative isobaric tags for relative and absolute quantitation (iTRAQ), and the whole protein profiling in immature (green) and mature (red) fruits was obtained. This has allowed identifying a total of 57 peroxisomal proteins from pepper fruits present in the matrix or membrane-bound. Furthermore, it was also found that some of these peroxisomal proteins were differentially expressed during the ripening process. To our knowledge, this is the first report about the analysis of the peroxisomal protein profile during the ripening of pepper fruits and provides the basement for future studies on the physiological relevance of peroxisomes in fruits.

## Materials and Methods

### Plant Material

Sweet California-type pepper (*Capsicum annuum* L., cv. Melchor) fruits were obtained from plants grown in plastic-covered greenhouses (Syngenta Seeds, Ltd., El Ejido, Almería, Spain). Fruits at two ripening stages were analyzed: immature green and ripe red. Fruits selected at each stage had similar phenotypic characteristics (size, shape, and color). In all cases, pericarp was prepared from, at least, four fruits (biological replicates) at each stage (one fruit per plant, five plants, each one having green and red fruits). After harvesting, fruits were cut into small cubes (5 mm/edge), frozen under liquid nitrogen, and then stored at –80°C until use.

### Fruit Sample Preparation for Whole Proteomic Analysis

Fruit samples (green and red, four replicates each) were ground under liquid nitrogen in a IKA A11 Basic mill and then dissolved in a ratio 1:1 (w:v) with 50 mM Tris-HCl buffer, pH 7.5, 0.1 mM EDTA, 0.1% (v/v) Triton X-100, 10% (v/v) glycerol, 5 mM DTT. The extract was filtered through two layers of nylon cloth and centrifuged at 27,000 *g* for 15 min. Proteins from the supernatant were precipitated with 70% (v/v) cold acetone at 4°C for 30 min. The mix was spun at 16,000 *g* for 15 min and the pellet was re-suspended into Tris-HCl 50 mM, pH 7.5 for 12–14 h at 4°C. Finally, samples were centrifuged at 39,000 *g* for 20 min, and the supernatants were recovered and cleaned by passing through PD-10© columns (General Electric). Eluted fractions from the columns were divided into 200 μg-protein aliquots and were lyophilized for isobaric tags for relative and absolute quantitation (iTRAQ^®^) analysis.

### Protein Digestion and Tagging With iTRAQ-8-plex^®^ Reagent

For digestion, 50 μg of protein from each pepper sample was precipitated using methanol/chloroform. Protein pellets were re-suspended and denatured in 20 μL of 6 M guanidine hydrochloride prepared in 100 mM HEPES, pH 7.5, reduced with 1 μL of 50 mM Tris (2-carboxyethyl)phosphine (TCEP, AB SCIEX), pH 8.0, at 60°C for 30 min, followed by the addition of 2 μL of 200 mM cysteine-blocking reagent (methyl methanethiosulfonate (MMTS, Pierce) for 10 min at room temperature. Samples were then diluted up to 120 μL to reduce guanidine concentration with 50 mM TEAB (tetraethylammonium tetrahydroborate). Digestions were initiated by adding 2 μg of sequence grade-modified trypsin (Sigma-Aldrich, Madrid, Spain) to each sample in a ratio of 1/25 (w/w), which were then incubated at 37°C overnight on a shaker. Sample digestions were evaporated to dryness. Each peptide solution was labeled at room temperature for 2 h with a half unit of iTRAQ Reagent Multi-plex kit (AB SCIEX, Foster City, CA, United States) previously reconstituted with 80 μL of 70% ethanol/50 mM TEAB. After labeling, samples were combined and labeling reaction was stopped by evaporation in a Speed Vac.

### Liquid Chromatography and Mass Spectrometry Analysis

A 2 μg aliquot of the iTRAQ-labeled mixture was subjected to nano-LC ESI-MS/MS analysis using a nano liquid chromatography system (Eksigent Technologies nanoLC Ultra 1D plus, AB SCIEX, Foster City, CA, United States) coupled to high-speed Triple TOF 5600 mass spectrometer (SCIEX, Foster City, CA, United States) with a Nanospray III source. The injection volume was 5 μL. The analytical column used was a silica-based reversed-phase Peptide BEH C18 column 75 μm × 15 cm, 1.7 μm particle size, and 130 Å pore size (Waters, Ireland). The trap column was an Acclaim PepMap 100 C18 ChromXP (Thermo Scientific, Madrid, Spain), 3 μm-particle diameters, 100 mm × 2 cm, switched on-line with the analytical column. The loading pump delivered a solution of 0.1% (v/v) formic acid in water at 2 μL/min. The nano-pump provided a flow-rate of 250 nL/min and was operated under gradient elution conditions, using 0.1% (v/v) formic acid in water as mobile phase A, and 0.1% (v/v) formic acid in acetonitrile as mobile phase B, under the following schema: isocratic conditions of 96% A: 4% B for 5 min, a linear increase to 40% B for 205 min, then a linear increase to 90% B for 15 additional minutes, isocratic conditions of 90% B for 10 min and return to initial conditions in 2 min. The total gradient length was 250 min.

Data acquisition was performed with a TripleTOF 5600 System. Ionization occurred under the following conditions: ion spray voltage floating (ISVF) 2800 V, curtain gas (CUR) 20, interface heater temperature (IHT) 150, ion source gas 1 (GS1) 20, declustering potential (DP) 85 V. All data were acquired using information-dependent acquisition (IDA) mode with Analyst TF 1.7 software (AB SCIEX, United States). For IDA parameters, 0.25 s MS survey scan in the mass range of 350–1250 Da was followed by 25 MS/MS scans of 150 ms in the mass range of 100–1500 (total cycle time: 4 s). Switching criteria were set to ions greater than mass to charge ratio (m/z) 350 and smaller than m/z 1250 with a charge state of 2–5 and an abundance threshold of more than 90 counts per second (cps). Former target ions were excluded for 20 s. IDA rolling collision energy (CE) parameters script was used for automatically controlling the CE.

### Database Search and Quantitative Analysis

MS/MS spectra were exported to Mascot generic format (MGF) using Peak View v1.2.0.3 and searched using Mascot Server 2.5.1, OMSSA 2.1.9, X!TANDEM 2013.02.01.1, and Myrimatch 2.2.140 against a composite target/decoy database built from the 38,628 *Capsicum annuum* bell pepper sequences at Uniprot (proteome ID UP000189700)^1^ plus some commonly occurring contaminants. Correctly identified peptides from an initial X! TANDEM search with a mass error tolerance of 35 ppm were used to recalibrate parent ion mass measurements in all spectra using linear models. All search engines were then configured to match potential peptide candidates to recalibrated spectra with a mass error tolerance of 10 ppm and fragment ion tolerance of 0.02 Da, allowing up to two missed tryptic cleavage sites and a maximum isotope error (13C) of 1. Fixed MMTS modification of cysteine and variable oxidation of methionine, pyroglutamic acid from glutamine or glutamic acid at the peptide N-terminus, and modification of lysine, tyrosine, and peptide N-terminus with iTRAQ 8-plex reagents were also considered. Score distribution models were used to compute peptide-spectrum match *p*-values (Ramos-Fernández et al., 2008), and spectra recovered by a False Discovery Rate (FDR) ≤ 0.01 (peptide-level) filter were selected for quantitative analysis. Differential regulation was measured using linear models (López-Serra et al., 2014), and statistical significance was measured using *q*-values (FDR).

For quantitative analysis of the sweet pepper fruit whole proteome iTRAQ was used due to its accuracy, discriminating capacity among treatments, and reproducibility. In fact, isobaric tagging quantification by mass spectrometry generally underestimates the magnitude of the differential abundance of proteins (DAP) due to contamination of quantitative signals with co-eluting background peptides, the so-called ratio compression effect, rendering fold change cutoff criteria useless. The strength of this compression effect is highly dependent on the tradeoff between sample complexity and data acquisition effort (peptide fractionation and gradient length), as well as instrument-specific factors. Furthermore, due to the stochastic nature of proteome sampling in quantitative shotgun proteomics approaches, data quality is highly variable across quantified proteins. Relatively low magnitude fold change values may achieve outstanding statistical significance if backed up by a sufficiently high number of peptides with good quality signals, with measured abundance ratios being consistently reproducible across biological replicates. On the other hand, high fold change proteins with very poor quality evidence rarely achieve low *q*-values. The log_2_-foldchange (log_2_FC) value represents the fold change of protein between two situations (A, green fruits; B, red fruits) and it is calculated according to the formula: log_2_FC = log_2_(B) – log_2_(A). All analyses were conducted using software from Proteobotics (Madrid, Spain).

### Functional Analysis

The different sets of obtained proteins were used to carry out functional analyzes by searching for enriched GO (Gene Ontology) and KEGG (Kyoto Encyclopedia of Genes and Genomes) terms, from different tools hosted on different web servers: AgriGO v2.0 (Tian et al., 2017), PlantRegMap (Jin et al., 2017), and KOBAS 3.0 (Xie et al., 2011). Finally, the GO terms that were significantly enriched were analyzed with REViGO (Supek et al., 2011) to eliminate redundant categories.

## Results

### Whole Proteome of Pepper Fruit During Ripening

**FIGURE 1:**
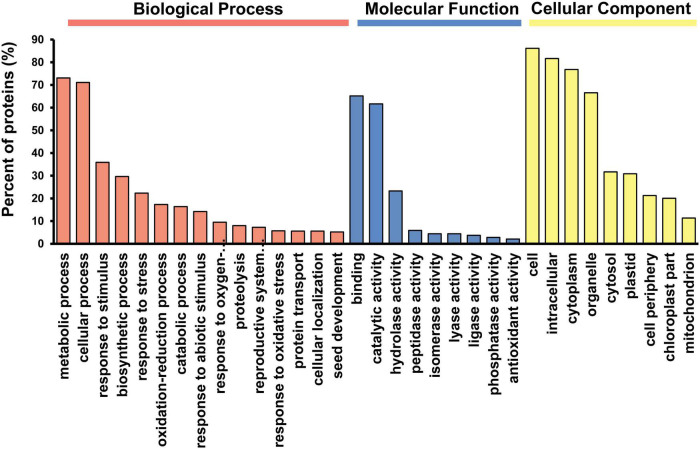
Distribution of the GO (Gene Ontology) categories assigned to the identified proteins from pepper fruit. Proteins were classified according to three categories: Biological Processes, Molecular Function and Cellular Components. After GO term enrichment analysis, the different categories with their corresponding *p*-values were filtered using the REViGO tool to remove redundant GO terms.

The analysis of pepper fruit whole proteome by the iTRAQ approach allowed the identification of a total of 2,284 proteins, of which 2,253 (98.6%) shared at least one orthologous sequence with *Arabidopsis thaliana.* With this information, an enrichment analysis was carried out in GO terms to determine the functional processes in which these proteins were involved. Among the different orthologs, 1,971 proteins were assigned to GO categories, with 1,331 of them (67.5%) being statistically enriched after analysis. Within the most significant GO terms 827, 340, and 163 proteins were grouped into the categories of biological processes, molecular functions and cellular components, respectively (Figure 1). Regarding the different categories belonging to biological processes, a high percentage of proteins were linked to metabolic and cellular processes. Likewise, it is worth highlighting other functions associated with response to different types of stress, along with other metabolic aspects involved in catabolism such as proteolysis. In this same group, a smaller number of proteins were related to the developmental processes of the reproductive system, highlighting the growth of some organs such as seeds. Among the different protein functions, the binding categories and different catalytic activities such as hydrolases, peptidases, isomerases, lyases, ligases and phosphatases stood out, and 2% of identified proteins were linked to antioxidant activities. Concerning the cell components, the majority of proteins were classified in the category corresponding to the cell, the intracellular space, and other subcellular locations such as the cytoplasm, plastids or mitochondria.

**FIGURE 2:**
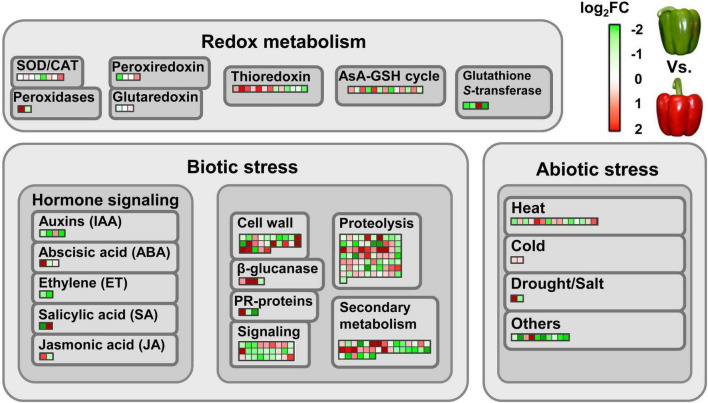
Differentially abundant proteins involved in redox metabolism and (a)biotic stress responses during the ripening sweet pepper fruits. Each colored square represents a protein. Green and red colors indicate down- and up-regulation at fruit ripening, respectively, according to log_2_FC values. FC, fold change. In the above right scale, red means log_2_FC > 0, whereas green means log_2_FC < 0. AsA-GSH cycle, ascorbate-glutathione cycle; CAT, catalase; PR-proteins, pathogenesis-related proteins; SOD, superoxide dismutase.

Within the set of identified proteins, it was estimated that around 50% of them modified their abundance due to the ripening process. Among the different quantified proteins, 692 (27%) were found to be significantly more abundant in the green fruits compared to red ones. On the contrary, 497 (19%) showed a higher abundance after the ripening process. To determine the different physiological events in which these proteins were involved, a functional analysis was carried out using MapMan software. Special attention was paid to those proteins related to redox metabolism and to response mechanisms against different types of stress (Figure 2). Thus, remarkable differences were found in the abundance of proteins that are part of complex antioxidant systems such as superoxide dismutases (SODs), catalase (CAT), peroxidases, peroxiredoxins and enzymes associated with the ascorbate-glutathione cycle (AsA-GSH), among others (Figure 2). Regarding the processes of response to abiotic stress, numerous proteins involved in hormonal signaling mechanisms were also found. Within this group, it is worth highlighting the regulation of those proteins involved in the synthesis of ethylene (ET), such as 1-aminocyclopropane-1-carboxylate (ACC) oxidase 4 (ACO4), whose relative abundance was higher in green fruits compared to red ones. Other affected metabolic categories were related to the maintenance of the cell wall structure, secondary metabolism, proteolysis and different signaling processes. Finally, a large group of proteins associated with different types of abiotic stress showed a greater abundance in green fruits compared to red ones. However, the levels of response proteins to cold stress were higher in red fruits.

The proteomic data were deposited in the repository PRIDE/ProteomeXchange with reference PXD010457.

### *In silico* Analysis of the Peroxisomal Proteome Mined From the Whole Fruit Proteome

**FIGURE 3:**
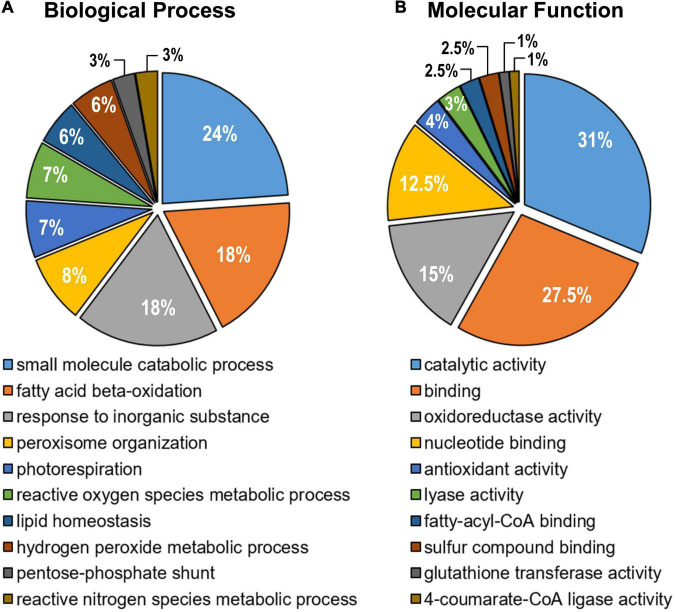
Gene Ontology enrichment analysis of the identified peroxisomal proteins from sweet pepper (*Capsicum annuum*, L.) fruit. **(A)** Biological process. **(B)** Molecular function. After GO term enrichment analysis, the different categories with their corresponding *p*-values were filtered using the REViGO tool to remove redundant GO terms. The number of proteins assigned to each functional category is expressed as a percentage (%).

Once the identifiers (Arabidopsis Genome Initiative –AGI- Locus Code) were obtained, these proteins were confronted with the most recently updated list of Arabidopsis peroxisomal proteins previously reported (Pan and Hu, 2018). This comparative analysis allowed identifying a total of 57 peroxisomal proteins. With this information, an enrichment analysis was carried out in GO terms to determine the functional processes where these peroxisomal proteins are involved (Figure 3). Among the different metabolic functions, it should be highlighted several catabolic processes such as the fatty acid β-oxidation, photorespiration and lipid homeostasis. Other proteins were involved in the structural organization of the peroxisome and associated with metabolic processes linked to reactive oxygen (ROS) and nitrogen (RNS) species (Figure 3A). Regarding the different molecular functions, the majority of peroxisomal proteins detected were related to catalytic activity and binding categories (Figure 3B).

A deeper analysis of the potential peroxisomal proteins from pepper fruits allowed classifying them according to their subcellular location and/or the absence/presence/type of peroxisomal targeting signals (PTSs). Table 1 shows the 44 identified peroxisomal matrix proteins containing canonical PTSs, with 36 proteins having a PTS type 1, and 8 containing a PTS type 2. In the same Table 1, their relative abundance, either upregulated, downregulated, or unaffected during sweet pepper fruit ripening, is also indicated. Table 2 shows the 5 peroxisomal matrix proteins lacking PTS, whereas Table 3 displays the 8 identified peroxisomal membrane proteins (PMPs). Consequently, this experimental approach using the iTRAQ-based protein profiling has increased the number of peroxisomal proteins of pepper fruits in comparison to our previous studies where, using pepper fruit peroxisomes purified by the combination of differential and sucrose density-gradient centrifugations and then subjected to 2-D electrophoresis and MALDI-TOF/TOF analyses, allowed to discriminate 39 polypeptides although only 13 out of them were identified with a high protein score (99%) confidence interval (C.I.) (Palma et al., 2018).

**TABLE 1:** Identified peroxisomal matrix proteins containing PTS which are differentially expressed during sweet pepper fruit ripening.

Protein name	Abbreviation	UniProt ID	PTS	log_2_FC	Regulation
**PTS1**					
Alanine-glyoxylate aminotransferase	AGT	A0A1U8ESR9	SRI	1.396	UP
Probable acyl-CoA dehydrogenase	IBR3	A0A2G2ZKA1	AKL	1.376	UP
2-hydroxyacyl-CoA lyase	HPCL2	A0A1U8FNI1	HKN^(1)^	1.109	UP
Acyl-coenzyme A oxidase 4	ACOX4	A0A1U8ERN1	SRL	0.906	UP
Monodehydroascorbate reductase 1	MDAR1	A0A1U8E6R6	SKI	0.972	UP
4-coumarate-CoA ligase 7	4CLL7	A0A2G2YHF4	SKL	0.793	UP
Carboxypeptidase	CP	A0A1U8H246	KNI^(1)^	0.894	UP
Glutamate-glyoxylate aminotransferase 2	GGAT2	A0A1U8EMR4	SRM	0.884	UP
Enoyl-CoA hydratase 2	ECH2	A0A1U8F2R5	SSL	0.595	UP
Glutathione reductase	GR2	A0A1U8G435	TNL	0.459	UP
Superoxide dismutase [CuZn]	SOD	A0A1U8EPJ0	SSV	0.655	UP
Amine oxidase	AOX	A0A1U8EUZ0	AKL	0.374	UP
Probable acetyl-CoA acetyltransferase	THIC2	A0A1U8GL52	SSL	1.302	UP
Hydroxypyruvate reductase	HPR	A0A1U8DY61	SKL	–	NONE
Sulfite oxidase	SO	A0A2G3ACR2	ANL	–	NONE
3-hydroxyacyl-CoA dehydrogenase	HADH	A0A1U8EU39	SRL	–	NONE
Dienoyl-CoA isomerase	DCI	A0A2G3ACC8	AKL	–	NONE
4-coumarate-CoA ligase 5	4CLL5	A0A2G2YA92	SKL	–	NONE
Acyl-coenzyme A oxidase 1	ACOX1	A0A2G3AEV5	ARL	–	NONE
Zinc-binding alcohol dehydrogenase domain-containing protein 2	ZBADH	A0A1U8DX36	AKL	–	NONE
Insulin-degrading enzyme-like 1	IDE1	A0A1U8GL11	VKL^(2)^	–1.689	DOWN
NADP-isocitrate dehydrogenase	ICDH	A0A2G2Y555	PKI^(2)^	–1.373	DOWN
6-phosphogluconate dehydrogenase 2	6PGDH2	A0A1U8F1E8	SKI	–0.017	DOWN
Isopentenyl-diphosphate isomerase	IDI2	A0A2G2ZKS5	HKL	–1.543	DOWN
(S)-2-hydroxy-acid oxidase	HAO	A0A1U8EZL8	PRL	–0.638	DOWN
NADH:ubiquinone reductase	NDB1	A0A1U8EN49	SRI	–1.147	DOWN
Isopentenyl-diphosphate isomerase	IDI1	A0A2G2Y814	HKL	–1.206	DOWN
Methyltetrahydropteroyltri glutamate-homocysteine S-methyltransferase^(3)^	METE1	A0A1U8F6S1	SAK	–0.899	DOWN
Urate oxidase	UO	A0A2G2Y6K0	SNM	–1.889	DOWN
Acyl-CoA thioesterase 2	ACTES	A0A1U8E2R5	PKL	–0.319	DOWN
4-coumarate-CoA ligase 4	4CLL4	A0A2G3AFX5	SKL	–	NONE
12-oxophytodienoate reductase 3	OPR3	A0A1U8H923	SRL	–	NONE
Probable 6-phosphogluconolactonase 4	6PGL4	A0A1U8FYP2	SKL	–	NONE
Uncharacterized protein	UP3	A0A2G2Z370	ASL	–	NONE
Catalase 2	CAT2	A0A1U8FMA2	QKL (internal)	–1.102	DOWN
Catalase 3	CAT3	A0A1U8GAA6	QKL (internal)	–1.374	DOWN
**PTS2**					
Acyl-coenzyme A oxidase 3	ACOX3	A0A1U8ENR3	RIx5HL	0.786	UP
Malate dehydrogenase	PMDH	A0A1U8FSV3	RIx5HL	1.333	UP
3-ketoacyl-CoA thiolase 2	ACAA1	A0A2G2ZTY4	RQx5HL	2.402	UP
Mevalonate kinase	MKV	A0A1U8GJW7	DVx5QM	–	NONE
Citrate synthase	CISY2	A0A1U8FK78	RLx5HL	–	NONE
Long chain acyl-CoA synthetase 6	ACLS	A0A2G2Y0B5	RLx5HL	–	NONE
Hydroxyisourate hydrolase^(4)^	HSH	A0A2G3ALZ5	RVx5HL	–	NONE
14 kDa zinc-binding protein^(4)^	ZBP	A0A1U8F6W1	RLx5HF	–	NONE

*^(1)^This putative PTS1 is experimentally not yet validated (Lingner et al., 2011).*

*^(2)^The Uniprot database assigns these proteins to be located in pepper peroxisome. The tripeptides VKL and PKI at the C-terminus have been experimentally verified (Lingner et al., 2011).*

*^(3)^The Uniprot database assigns the Arabidopsis ortholog protein is protein to be located in peroxisome.*

*^(4)^The Uniprot database assigns this protein to be located in rice peroxisome.*

**TABLE 2:** Peroxisomal matrix protein lacking PTS which are differentially expressed during sweet pepper fruit ripening.

Protein name	Abbreviation	UniProt ID	log_2_FC	Regulation
Serine/threonine-protein phosphatase 2A	STP2A	A0A1U8EKG3	1.531	UP
Serine/threonine-protein phosphatase	STP	A0A2G2YDX1	–	NONE
Probable sarcosine oxidase	PIPOX	A0A1U8F0Q0	–0.644	DOWN
Nucleoside diphosphate kinase	NDK1	A0A2G3AM91	–0.456	DOWN
Glyoxalase 1	GLX1	A0A2G3A977	–0.915	DOWN

**TABLE 3:** Peroxisomal membrane-associated proteins (PMPs) which are differentially expressed during sweet pepper fruit ripening.

Protein	Abbreviation	UniProt ID	log_2_FC	Regulation
Peroxisome biogenesis protein 19	PEX19	A0A2G3AER5	1.084	UP
Fission 1 protein	FP1	A0A1U8FBV7	0.669	UP
ABC transporter D 1	ABCD1	A0A2G2Y840	0.724	UP
Peroxin 4	PEX4	A0A1U8EYW8	–	NONE
Peroxisome biogenesis protein 22	PEX22	A0A1U8F7K6	–	NONE
L-ASCORBATE PEROXIDASE 5	APX5	A0A2G2Y2S6	–	NONE
Ras-related protein RABE1C	RABE1C	A0A1U8GLM9	–	NONE
Dehydroascorbate reductase 2	DAR2	A0A1U8GZY4	–0.662	DOWN

### Modulation of Peroxisomal Proteins During Pepper Fruit Ripening

**FIGURE 4:**
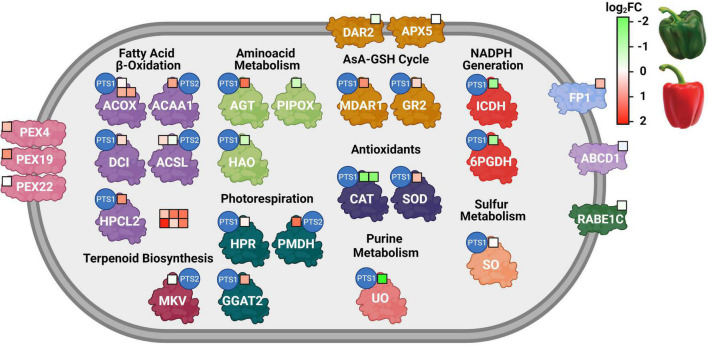
Regulation of peroxisomal proteins during sweet pepper fruit ripening. Each colored square represents a protein. Green and red colors indicate down- and up-regulation at fruit ripening, respectively, according to log_2_FC values. 6PGDH, 6-phosphogluconate dehydrogenase; ABCD1, ABC transporter D 1; ACAA1, 3-ketoacyl-CoA thiolase; ACOX, acyl-CoA oxidase; ACSL, long-chain acyl-CoA synthetase; AGT, alanine:glyoxylate aminotransferase; APX5, L-ascorbate peroxidase 5; CAT, catalase; DCI, dienoyl-CoA isomerase; FP1, fission protein 1; GGAT2, glutamate glyoxylate aminotransferase 2; GR, glutathione reductase 2. HAO, (S)-2-hydroxy-acid oxidase; HPCL2, 2-hydroxyacyl-CoA lyase; HPR, hydroxypyruvate reductase; ICDH, isocitrate dehydrogenase; MDAR1, monodehydroascorbate reductase 1; MKV, mevalonate kinase; PEX, peroxin; PIPOX, probable sarcosine oxidase; PMDH, malate dehydrogenase 1; RABE1C, Ras-related protein RABE1C; DAR2, dehydroascorbate reductase 2; SOD, superoxide dismutase; UO, urate oxidase. FC, fold change. Red means log_2_FC > 0, green means log_2_FC < 0.

Besides the identification of the peroxisomal proteome, the used experimental approach (iTRAQ) also allowed to compare and quantify the relative abundance of these proteins at the immature (green) and ripe (red) stages. Figure 4 depicts the biological process of each of these peroxisomal proteins and its corresponding heat map regulation during ripening from green to red. Thus, among the 35 identified proteins with showed significant differences in their abundance during ripening, 19 proteins were overexpressed, 16 were repressed, and the other 22 peroxisomal proteins were unaffected. Thus, it can be seen in Figure 4 and Tables 1-3 that most of the proteins linked to the β-oxidation process, together with the ABCD1 transporter responsible for the import of fatty acids into the peroxisome, showed a higher abundance in red fruits. This same trend was found in the peroxins PEX4 and PEX19. Regarding the antioxidant systems, different components of the ascorbate-glutathione cycle were detected, among which the overexpression of two isoenzymes (MDAR1 and GR2) stand out. In this same group, we found the CAT3 isoenzyme, with higher levels of abundance in green peppers, while a CuZn-SOD isoenzyme displayed the opposite pattern. In addition, the expression of two enzymes that generate reducing power in the form of NADPH (6PGDH and NADP-ICDH) decreased during maturation.

## Discussion

Proteomic studies on fruits are relatively scarce compared to other plant organs such as cotyledons, roots or leaves (Palma et al., 2018). However, the technological advances associated with the proteomic field have led to a real scientific revolution in the analysis of different biological processes, including fruit ripening and senescence (Palma et al., 2011; Wu et al., 2017). In recent years, two-dimensional electrophoresis (2-DE) has been the most widely used protein separation method in proteomic analyses, carrying out extensive research on the expression and accumulation of proteins in different fruits such as apples (Marondedze and Thomas, 2012; Sánchez-Bel et al., 2012; Zheng et al., 2013), tomatoes (Rocco et al., 2006; Faurobert et al., 2007; Choi et al., 2022), grape (Sarry et al., 2004), or peaches (Prinsi et al., 2011; Zhang et al., 2011). However, these studies have certain limitations that are intrinsic to the methodology used, such as its low reproducibility, the difficulty in separating hydrophobic proteins, the low number of identified proteins, as well as the low solubility of certain proteins due to their physical-chemical properties (Görg et al., 2004; Magdeldin et al., 2014). Alternatively, the use of iTRAQ^®^ involves several advantages in proteomic analyses, including the conservation of information related to post-translational modifications (PTMs) (Chaube, 2014).

### During Fruit Ripening, the Whole Proteome Shows a Remarkable Modulation of Proteins Involved in Redox Metabolism and (A)biotic Stress Responses

In our experimental sweet pepper fruit model, the proteomic analysis allowed the identification of a total of 2,284 proteins. This value was higher than the proteins found in similar studies in other fruits such as grapes (674; Kambiranda et al., 2014) or 1,384 in peaches (Wu et al., 2017); but also somehow lower than those identified in tomato with 2,336 proteins (Liu et al., 2016) or 5,578 in melon (Chen et al., 2021). Likewise, the functional annotation of protein relative abundance in GO terms was similar to those described in other fruits of agronomic interest such as tomatoes (Liu et al., 2016) or grapes (Kambiranda et al., 2014). It is remarkable that above the half of the identified proteins was modulated during ripening. The identification of numerous proteins involved in redox homeostasis represents new evidence of the importance of oxidative metabolism during the ripening of sweet pepper fruit. In addition, different proteins related to hormonal signaling mechanisms were found. Among them, it is worth highlighting the indoleacetic acid (IAA) amido hydrolase enzymes, responsible for controlling the levels of IAA in plant cells by converting auxin-amino acid conjugates into free IAA molecules (Bartel and Fink, 1995; LeClere et al., 2002). Although it was thought that this was their only function, it has recently been shown that these proteins can regulate the expression of the enzyme 1-aminocyclopropane-1-carboxylic (ACC) synthase (ACS), altering the synthesis of ethylene during peach ripening (Wang et al., 2021). This would imply a new point of interaction between auxin metabolism and ET, whose possible implications in pepper fruit ripening could be interesting to address in future research. On the other hand, the abscisic acid (ABA) seems to play a fundamental role in the ripening of non-climacteric fruits. In this sense, we found a greater accumulation in red fruits of an enzyme involved in their biosynthesis, specifically an aldehyde oxidase (AO4). Its respective orthologous protein is involved in the senescence delay in Arabidopsis siliques through the oxidation of different aldehydes of a toxic nature, so that in pepper fruits it could have similar functions (Srivastava et al., 2017). Although the ripening of the pepper fruit has been classified as non-climacteric, it was observed a greater abundance in green fruits of the enzyme ACC oxidase (ACO), which is responsible for catalyzing the last step in the synthesis of ET (Houben and Van de Poel, 2019; Pattyn et al., 2021), which would indicate that, regardless of the physiological response of the fruits, this phytohormone could play essential roles in the ripening process (Bapat et al., 2010; Hou et al., 2018).

During ripening, a series of changes take place in fruits such as the accumulation of pigments, volatile compounds, organic acids and sugars that, added to different alterations in the firmness and texture of the fruits, constitute evolutionary strategies aimed at attracting animals that could help to seed dispersion. A considerable part of these modifications is dependent on secondary metabolism, which implies that during the ripening process different patterns of the enzymes involved in their metabolic pathways are produced. Therefore, the over-accumulation observed in red pepper fruits of enzymes involved in the synthesis of carotenoids such as phytoene desaturase (PDS), zeta-carotene desaturase (ZDS1), and lycopene β-cyclase (LCY1) (Hornero-Méndez et al., 2000; Berry et al., 2019) is not surprising. On the other hand, certain processes related to the growth and consistency of the fruits are determined by different modifications associated with the cell wall. In fact, several proteins related to the maintenance and syntheses of the different components that make up this structure were found (Brownleader et al., 1999; Paniagua et al., 2014; Forlani et al., 2019).

Throughout fruit development, a greater accumulation of proteins linked to different defense processes was also observed, including glucan endo-1,3-β-D-glucosidase (GLB) that could play important roles in protection against pathogens in sweet pepper, as has been proposed for other types of fruit (Prinsi et al., 2011; Kambiranda et al., 2014). Thus, these series of changes that occur during the ripening of the fruits imply an exhaustive control of the mechanisms associated with protein degradation and synthesis, as it has been observed in sweet pepper fruits (Palma et al., 2011). Despite their categorization as response proteins against different types of abiotic stress, many of these proteins participate in cell signaling processes through mechanisms of interaction with different phytohormones (Ku et al., 2018). Thus, the possible implications of these proteins during the ripening of the pepper fruit should be analyzed more exhaustively in future studies.

### Scrutiny of Peroxisomal Proteome Mining From the Whole Fruit Proteome

Peroxisomes remain one of the least studied organelles in plant biology and the information available on their proteome in fruits is virtually very limited (Palma et al., 2018). There are few biochemical characterizations of peroxisomes from some fruits such as pepper (Mateos et al., 2003; Palma et al., 2015, 2018; Rodríguez-Ruiz et al., 2019), olive (López-Huertas and del Río, 2014), and apple (Zarei et al., 2015). And, there are only available few proteome studies from isolated peroxisomes, i.e., from etiolated soybean cotyledons (Arai et al., 2008), leaf peroxisome from spinach leaves (Babujee et al., 2010) and etiolated Arabidopsis seedlings (Quan et al., 2013). At present, the best characterized peroxisomal proteome has been obtained from *Arabidopsis thaliana* leaves using different technical approaches and under different events (Fukao et al., 2002; Reumann, 2004; Reumann et al., 2004, 2007, 2009; Pan and Hu, 2018; Pan et al., 2018, 2019), including some novel machine learning prediction methods (Lingner et al., 2011; Reumann et al., 2016; Reumann and Chowdhary, 2018). To our knowledge, the most recent peroxisomal proteome in Arabidopsis is constituted of 199 proteins, including 144 targeting signal (PTS)-containing matrix proteins, 45 membrane proteins, and 10 proteins lacking recognizable PTS (Pan et al., 2018). This information from Arabidopsis peroxisomes has been very valuable for our mining study and has allowed identifying in the pepper fruit proteome obtained by iTRAQ a total of 57 peroxisomal proteins located in the matrix and membrane, and the majority harboring either a PTS1 or PTS2. Although some of the assigned putative PTS1 (HKN and KNI) have experimentally not yet been validated (Lingner et al., 2011; Wang et al., 2017), it cannot be discarded the occurrence of a “piggyback import” mechanism which could occur in the PTS1 and PTS2 import pathway where a protein without a PTS can dimerize with a PTS-cargo protein and then both proteins could be imported into the peroxisome (Cross et al., 2016).

Peroxisomes have an essentially oxidative metabolism and constitute one of the main sources of intracellular H_2_O_2_ production in plant cells (Corpas, 2015; del Río and López-Huertas, 2016; Corpas et al., 2017, 2020). Therefore, a considerable number of the identified proteins in pepper peroxisomes belongs to different antioxidant systems devoted to maintaining the ROS homeostasis previously described in peroxisomes from different plant species, and it includes catalase (Palma et al., 2020a), CuZn-SOD (Bueno et al., 1995; Corpas et al., 1998b; Palma et al., 2018), and all the enzymatic components of ascorbate-glutathione cycle. The enzymatic members of this cycle are located either in the peroxisomal matrix, such as glutathione reductase (GR) and monodehydroascorbate peroxidase (MDAR), and bound to the membrane, as it has been reported for ascorbate peroxidase (APX) and dehydroascorbate reductase (DAR) (Corpas et al., 1994; Bunkelmann and Trelease, 1996; Jiménez et al., 1997; Nito et al., 2001; Leterrier et al., 2005; Narendra et al., 2006; Romero-Puertas et al., 2006; González-Gordo et al., 2022a). Furthermore, the presence of some components of the pentose phosphate pathway involved in the generation of NADPH reported here is also in good agreement with previous reports in pea and Arabidopsis peroxisomes (Corpas et al., 1998a; Corpas et al., 1999; Fernández-Fernández and Corpas, 2016; Hölscher et al., 2016; Lansing et al., 2020). Thus, the differential abundance of the antioxidant systems throughout ripening suggests that peroxisomes may play an important role during this physiological process. Additionally, the NADP-isocitrate dehydrogenase (NADP-ICDH) catalyzes the oxidative decarboxylation of L-isocitrate to 2-oxoglutarate with the production of the reduced coenzyme NADPH, which is an essential component in the cellular redox homeostasis. Its activity has been found in purified peroxisomes from pepper fruits (Mateos et al., 2003) and, among the four NADP-ICDHs detected in pepper fruit proteome, one has the tripeptide PKI as PTS1 (Lingner et al., 2011). Nevertheless, this enzyme has been also characterized in peroxisomes from pea during leaf senescence (Corpas et al., 1999) and from Arabidopsis leaves being involved in the stomatal closure (Leterrier et al., 2016). In the case of Arabidopsis, the peroxisomal NADP-ICDH (At1g54340) has the tripeptide SRL which is a canonical PTS1 found also in the NADP-ICDH from *Camelina sativa* (XP_010480188.1), *Brassica rapa* (RID54472.1) or *Eutrema salsugineum* (XP_024005998.1).

In-plant peroxisomes, there are several NADPH-consuming enzymes such as 2,4-dienoyl-CoA reductase which participates in the degradation of unsaturated fatty acids; nitric oxide (NO) synthase-like activity which generates the signal molecule NO; glutathione reductase that regenerates the antioxidant GSH; 12-oxophytodienoate reductase (OPR3) which is involved in jasmonic acid (JA); nudix hydrolase 19 (NUDX19) which regulates the NADPH content (Corpas and Barroso, 2018 and references therein); and very recently the benzaldehyde synthase that generates benzaldehyde which is considered the simplest aromatic aldehyde that has multiple functions in plants such as a pollinator attractant, flavor volatile and antifungal compound (Huang et al., 2022).

Other representative metabolic pathways of peroxisomes are the photorespiration and the β-oxidation (Baker et al., 2006; Hu et al., 2012). Apart from their catabolic function, the compounds derived from the β-oxidation participate in the control of a wide variety of cellular processes through their interaction with phytohormones such as jasmonic acid (JA), indole acetic acid (IAA) (Nyathi and Baker, 2006; Gfeller et al., 2010; León, 2013; Spiess and Zolman, 2013; Wasternack and Strnad, 2018; Corpas et al., 2019), or salicylic acid (SA) (Chaouch and Noctor, 2010; Chaouch et al., 2010; Corpas and Barroso, 2018). The identified enzymes in our analysis that participate in the β-oxidation showed a higher accumulation in red peppers, suggesting that they could help in the hormonal regulation of the fruit ripening. At the same time, it was also found an increase in the content of proteins related to the division and biogenesis of the peroxisomes, such as the peroxisomal fission protein (FP1), and different peroxins (PEX) such as PEX4, PEX19, and PEX22 (Kao et al., 2018). This occurrence could lead to an increase in the number of peroxisomes in red pepper fruits, which would constitute a mechanism to struggle the nitro-oxidative stress associated with the ripening process (Chaki et al., 2015; Corpas et al., 2018; Rodríguez-Ruiz et al., 2019; González-Gordo et al., 2020).

Among the identified peroxisomal proteins in pepper fruits, some proteins can be cataloged as unexpected of this organelle, including the 4-coumaroyl-CoA ligase (4-CL), sarcosine oxidase (SOX), urate oxidase (UO), sulfite oxidase (SO), 5-methyltetrahydropteroyltriglutamate-homocysteine S-methyltransferase (METE1), and 12-oxophytodienoate reductase 3 (OPR3).

The 4-coumaroyl-CoA ligase (4-CL) participates in the biosynthesis of a diverse array of plant natural phenylpropanoid products (Dixon and Paiva, 1995; Rastogi et al., 2013; Jang et al., 2015), being the third enzyme of the phenylpropanoid pathway which drives the biosynthesis of compounds such as lignins, flavonoids, and coumarins (Muro-Villanueva et al., 2019; Dong and Lin, 2021). Specifically, the 4-CL catalyzes the generation of the coenzyme A thioester of cinnamates such as 4-coumaric, caffeic, and ferulic acids (Liu et al., 2017). Very recently, it has been demonstrated in Arabidopsis that the 4-coumaroyl-CoA ligase 8 (4-CL8) isozyme (At5g38120) is located in leaf peroxisomes and participates in the β-oxidative conversion of *p*-coumarate into 4-hydroxybenzoate, this one is implicated in the biosynthesis of the ring precursor of ubiquinone (Soubeyrand et al., 2019). Our proteomic identification of the peroxisomal 4-CL5 (A0A1U8F3W0) and 4-CL7 (A0A1U8ELY2), both containing the PTS1 sequence SKL, and having 87 and 77% similarities with the Arabidopsis 4-CL5 and 4-CL8, respectively, opens a new implication of fruit peroxisome in phenylpropanod pathway. Thus, the biosynthesis of lignin is needed for the firmness of some fruits. And flavonoids, such as anthocyanins, which have also antioxidant properties, may affect pigmentation and disease resistance in fruits (Singh et al., 2010; Wang et al., 2010, 2020). On the other hand, phenylpropanoids are also precursors of capsaicin and other capsaicinoids, alkaloids present exclusively in *Capsicum* species, which provides to their fruits the typical pungency trait (Kang et al., 2005; Nuñez-Palenius and Ochoa-Alejo, 2005; Materska and Perucka, 2010; Kehie et al., 2015; Palma et al., 2020b).

The peroxisomal ABC transporter of Arabidopsis ABCD1 is also indirectly related to the biosynthesis of several secondary metabolites (Biała and Jasiński, 2018). Among these molecules it can be highlighted: benzoic acids, possibly due to the transport of cinnamic acid/cinnamoyl-CoA into the peroxisome (Bussell et al., 2014); ubiquinone, as a consequence of the import of 4-coumarate/coumaroyl-CoA (Block et al., 2014); and flavonoids, through the facilitation of fatty acids breakdown, which induces flavonoid biosynthetic enzymes (Carrera et al., 2007).

Sarcosine oxidase (SOX) is a flavoprotein that catalyzes the oxidative demethylation of sarcosine to glycine, H_2_O_2_, and the cofactor 5,10-methylenetetrahydrofolate, which participate as an intermediary in the detoxification of formaldehyde. This enzyme is well known in animals and bacteria, it has been reported in Arabidopsis peroxisomes (Goyer et al., 2004), and its presence in pepper fruit peroxisomes is another unexpected event that opens new questions about its potential function in the ripening process.

Urate oxidase (UO) is part of the purine metabolism and catalyzes the conversion of uric acid to H_2_O_2_ plus 5-hydroxyisourate which is spontaneously transformed to allantoin plus CO_2_. The presence of this enzyme in peroxisome is well known for a long time but it has been associated with nitrogen metabolism (Hauck et al., 2014). However, UO regulates the uric acid content which has been detected in pea peroxisomes (Corpas et al., 1997) and is also an effective peroxynitrite (ONOO^–^) scavenger (Alamillo and Garcia-Olmedo, 2001). Peroxynitrite is generated by the interaction between superoxide (O_2_^•–^) and nitric oxide (NO) radicals, and its presence in Arabidopsis peroxisomes has also been reported (Corpas and Barroso, 2014). In the case of bell pepper fruits, the decrease in UO activity observed during ripening might involve an increase in uric acid levels in the peroxisomes of red fruits. This could be a defense mechanism to mitigate the harmful effects of the nitro-oxidative burst that occurs during fruit development (Corpas et al., 2018; Corpas and Palma, 2018).

Sulfite oxidase (SO) catalyzes the conversion of sulfite to sulfate plus H_2_O_2_ (Hänsch et al., 2007), and its occurrence in Arabidopsis peroxisomes has been also described (Hänsch, and Mendel, 2005; Hänsch et al., 2006; Byrne et al., 2009). It has been suggested that the potential function of this enzyme is to protect catalase activity since a low concentration of sulfite can inhibit the activity of this peroxisomal enzyme (Veljović-Jovanović et al., 1998). Being a key enzyme in sulfur metabolism (Randewig et al., 2012; Oshanova et al., 2021), SO has been also associated with the mechanism of response to drought stress in maize, since it seems to be involved in stomatal regulation and GSH-dependent antioxidant systems (Corpas and Barroso, 2015; Xia et al., 2018).

The 5-methyltetrahydropteroyltriglutamate-homocysteine *S*-methyltransferase (METE1) catalyzes the conversion of 5-methyltetrahydropteroyltri-L-glutamate plus L-homocysteine to tetrahydropteroyltri-L-glutamate plus L-methionine, which is an S-containing proteinogenic amino acid (Hesse and Hoefgen, 2003). The identified pepper METE1 has the tripeptide SAK as PTS1 which is identical to its Arabidopsis ortholog (At5g17920) found in several Arabidopsis peroxisomal proteomes (Reumann et al., 2009; Quan et al., 2013; Pan and Hu, 2018). However, its function in peroxisomes is still unknown and its role in fruits needs to be investigated further.

The enzyme OPR3 is a flavoprotein that required NADPH such as it has be mentioned. It catalyzes the reduction of the cyclopentenone ring of (9S,13S)-12-oxophytodienoate [(9S,13S)-OPDA] and is involved in the biosynthesis of jasmonic acid (JA) (Schaller et al., 2000). In tomato plants, its implication in the mechanism of defense against necrotrophic pathogens has been shown (Scalschi et al., 2015), but no reports on its function in the metabolism of fruit peroxisomes are available so far. The presence of OPR3 in peroxisomes from pepper fruits point toward the potential involvement of JA in the ripening of non-climacteric fruits. This issue, in conjunction with the profile of other phytohormones, is also a subject to be addressed in the future.

## Conclusion

**FIGURE 5:**
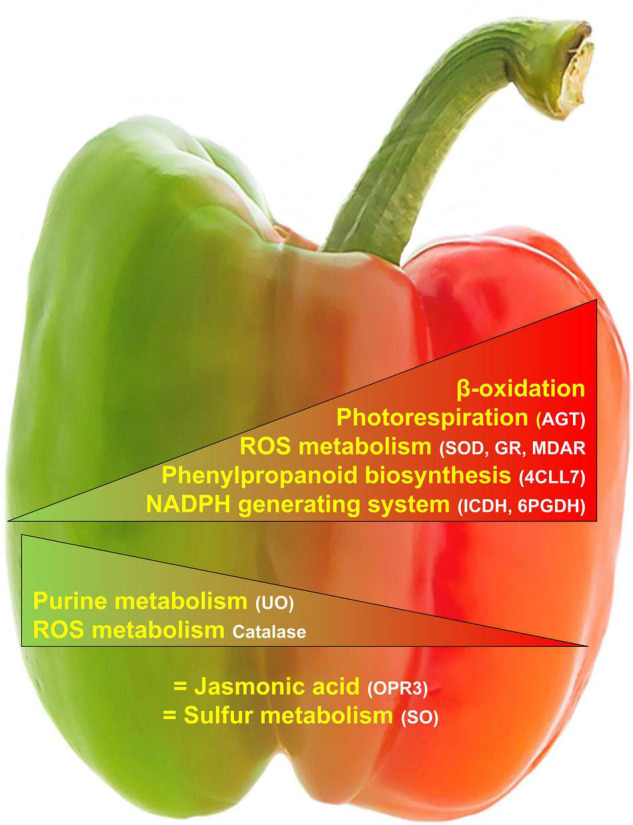
Working model of the main peroxisomal processes where the detected proteins are either positive/negatively modulated or not affected during pepper fruit ripening. 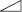, Upregulated. 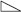, Downregulated. = , No change. 4CLL7, 4-coumarate-CoA ligase 7; 6PGDH, 6-phosphoglutamate dehydrogenase; AGT, Alanine-glyoxylate aminotransferase; GR, glutathione reductase; ICDH, isocitrate dehydrogenase; MDAR, monodehydroascorbate reductase; OPR3, 12-oxophytodienoate reductase 3; SOD, superoxide dismutase; UO, urate oxidase.

This new piece of information on the peroxisomal pepper obtained by mining the iTRAQ fruit proteome and the bioinformatics comparison with the already known Arabidopsis peroxisomal proteome has allowed advancing into the complex organization of metabolic pathways undergoing in fruit peroxisomes. Thus, besides the confirmation of some common enzymes already known to be present in peroxisomes from different plant organs (leaves or cotyledons), such as those involved in fatty acid metabolism, photorespiration, antioxidants, jasmonic, and auxin metabolism, there are unexpected enzymes, not reported earlier, to our knowledge, in fruit peroxisomes. A remarkable example is the presence of a 4-coumarate-CoA ligase whose its substrate, 4-coumaroyl CoA, is considered to be a cornerstone in the central phenylpropanoid biosynthesis in higher plants (Vogt, 2010). Figure 5 shows a working model of peroxisomal processes which are modulated during pepper fruit ripening. The provided data open new perspectives to investigate the function of peroxisomes in the ripening of fruits, an issue that should be explored to enrich our knowledge of the metabolic potentialities of peroxisomes in different physiological processes and fruit homeostasis. Although the data obtained constitute an advance in our knowledge of the peroxisomes of the pepper fruits, it should be highlighted that there are still many gaps related to peroxisomal components among the different plant organs and species that need to be deciphered.

## Data Availability Statement

The datasets presented in this study can be found in online repositories. The names of the repository/repositories and accession number(s) can be found in the article/supplementary material.

## Author Contributions

SG-G carried comparative analyses of the different iTRAQ database of sweet pepper fruits. FJC and JMP designed the work, drove and coordinated the tasks, and wrote the manuscript. All authors contributed to the article and approved the submitted version.

## Conflict of Interest

The authors declare that the research was conducted in the absence of any commercial or financial relationships that could be construed as a potential conflict of interest.

## Publisher’s Note

All claims expressed in this article are solely those of the authors and do not necessarily represent those of their affiliated organizations, or those of the publisher, the editors and the reviewers. Any product that may be evaluated in this article, or claim that may be made by its manufacturer, is not guaranteed or endorsed by the publisher.

## Footnotes

1
https://www.uniprot.org/proteomes/UP000189700
